# Nail alterations as a surrogate marker for the efficacy of low-dose metronomic chemotherapy

**DOI:** 10.3892/ol.2013.1178

**Published:** 2013-02-05

**Authors:** KAYOKO KIBATA, TAKESHI TAMAKI, NORIKO INAGAKI, MAKOTO OGATA, TOSHIKI SHIMIZU, SHOSAKU NOMURA

**Affiliations:** First Department of Internal Medicine, Kansai Medical University, Moriguchi, Osaka 570-8506, Japan

**Keywords:** docetaxel, nail alterations, onycholysis, subungual hyperkeratosis, metronomic chemotherapy, non-small cell lung cancer

## Abstract

Docetaxel is a well-known causative agent of nail alterations. The aim of this study was to reveal the impact of nail alterations associated with low-dose metronomic (LDM) docetaxel chemotherapy on the survival of non-small cell lung cancer (NSCLC) patients. Clinical information, survival data and nail alterations in patients treated with LDM docetaxel chemotherapy (docetaxel 15 mg/m^2^ per week) were retrospectively reviewed. Forty-nine patients were included in this study. Various nail alterations were observed in 17 of the 49 patients (34.7%). Onycholysis and subungual hyperkeratosis were observed in 22.4% and 10.2% of patients, respectively. The number of docetaxel administration cycles was correlated with the incidence and severity of nail alterations. Univariate and multivariate analysis clearly demonstrated that the occurrence of nail alterations was an independent favorable prognostic factor for overall survival. Nail alterations associated with treatment may act as a surrogate marker for the efficacy of low-dose metronomic docetaxel chemotherapy.

## Introduction

Systemic antitumor chemotherapy induces treatment-associated adverse effects in various organs, including the skin and nails (1). To date, skin and nail alterations have attracted a great deal of interest due to their potential as surrogate markers for molecular-targeted therapies, in particular epidermal growth factor receptor (EGFR)-targeted treatment (2). A close correlation has been observed between the severity of skin/nail alterations, treatment efficacy and the survival benefits of EGFR-targeted treatment (3).

An alternative approach to systemic chemotherapy is low-dose metronomic (LDM) chemotherapy, which is focused on the treatment of various neoplastic diseases (4–6). LDM chemotherapy involves frequent administration of a cytotoxic drug in a low dose to exert an alternative antitumor effect, in addition to a direct cytotoxic effect. More specifically, studies have revealed that LDM chemotherapy exerts an antitumor effect via vascular mechanisms (7–9).

Docetaxel, a semi-synthetic taxoid drug made from a precursor extracted from the needles of the European yew, *Taxus baccata*, enhances microtubule assembly and is recognized as an active metronomic agent. LDM docetaxel chemotherapy was used in a pilot study as a therapy for previously treated non-small cell lung cancer (NSCLC) (10). Docetaxel is also a well-known causative agent of nail alterations (11–13). Therefore, we hypothesized that nail alterations associated with treatment may be a surrogate marker for the efficacy of LDM docetaxel chemotherapy. In this study, we retrospectively analyzed the correlation between nail alterations and survival benefit. The results presented in this study demonstrate the favorable impact of treatment-associated nail alterations during LDM docetaxel chemotherapy on survival.

## Patients and methods

### Data collection

The medical records of all NSCLC patients who were treated between January 1999 and December 2010 in Kansai Medical University Takii Hospital (Moriguchi-City, Japan) were retrospectively reviewed after Institutional Review Board approval. Patients were included in this study if they had advanced stage (III or IV) or relapsed NSCLC that was treated with LDM chemotherapy using docetaxel. A clinical stage was assigned according to the sixth edition of the TNM Classification for Lung Cancer (14). Data with regard to gender, age, clinical stage, histological typing of cancer, Eastern Cooperative Oncology Group (ECOG) performance status (PS), progression-free survival (PFS) and overall survival (OS) were obtained retrospectively from the medical records. Adverse effects on the finger- and toenails observed during the treatment period were also ascertained retrospectively from the medical records and photographs of the nails of patients were retrieved with informed consent. All patients provided written informed consent before undergoing chemotherapy. The study was carried out according to the Declaration of Helsinki and was approved by the Institutional Ethics Review Board (Subcommittee for the Epidemiologic Study, Institutional Review Board of Kansai Medical University).

### Treatment

Docetaxel (15 mg/m^2^) diluted with 250 ml of a 5% glucose solution was administered intravenously over 60 min each week. The treatment was administered on a weekly basis without any intervals. To prevent a hyper-sensitive reaction, 2 mg of dexamethasone or H1-blocker was administered as a premedication to all patients. The treatment was repeated, with the exception of cases where disease progression was observed. Responses were evaluated using a chest CT every month during the treatment period. Administration of docetaxel was bypassed if the patient experienced unacceptable toxicity (e.g., grade 3 or worse hematological/non-hematological toxicity, excluding appetite loss, constipation and nausea/vomiting). Patients who refused to continue this regimen and those who showed a decline in performance status (PS; PS 4) were also allowed to avoid LDM chemotherapy.

### Statistical analysis

Statistically significant differences between groups were compared using Student’s t-test, a Chi-square test or Fisher’s exact test. Spearman’s correlation coefficients (two-tailed) were used to evaluate whether the number of docetaxel administration cycles correlated with the level of nail alterations. Logistic regression was used to test the univariate dose-response correlation and multivariate associations with the development of nail alterations. Variables were considered for the multivariate models if their univariate P-value was <0.25. Overall survival (OS) was defined as the time from the start of LDM chemotherapy to the time of death from any cause or to the date the patient was last known to be alive. Progression-free survival was defined as the time between the start of LDM treatment and disease progression, death or last known follow-up. Objective tumor responses to chemotherapy were evaluated using the Response Evaluation Criteria in Solid Tumors version 1.0 (15). The objective response rate (ORR) was defined as the number of patients showing a complete response (CR) and a partial response (PR) relative to the total number of patients evaluated. The disease control rate (DCR) was defined as the number of patients showing CR, PR and stable disease (SD) relative to the total number of patients evaluated. The minimum time interval between the 2 measurements required for the determination of SD was 6 weeks. The 95% confidence intervals (95% CI) for the ORR and DCR were calculated using binomial distribution. Univariate and multivariate analysis of PFS and OS were performed using the Kaplan-Meier product-limit method using the log-rank test and the Cox proportional hazards model, respectively. The 95% CI for the survival rate was calculated using Greenwood’s method. To calculate the 95% CI of the median survival time (MST), the Brookmeyer and Crowley method was used.

All statistical analysis was conducted using the JMP (version 9.0.2) software program for Windows (SAS Institute Inc., Cary, NC, USA). All statistical tests were two-sided and P<0.05 was considered to indicate a statistically significant difference.

## Results

### Patient characteristics

Between January 1999 and December 2010, 49 patients with NSCLC who met the eligibility criteria were enrolled in this study. Patient characteristics are summarized in Table I. All patients were Japanese and the median age was 69 years (range, 32–85 years). Ten patients were female and 39 were male. Adenocarcinoma and squamous cell carcinoma were present in 30 and 19 patients, respectively. Twenty-two patients had stage III disease, whereas 27 patients had stage IV disease. The ECOG PS was 0 and 1 in 21 and 28 patients, respectively. Twenty-seven patients had received at least 1 regimen of cytotoxic chemotherapy prior to this LDM regimen, but the remaining 22 patients had never been treated. The median number of cycles administered to the 49 patients was 16 (range, 2–60). Eleven patients received at least 1 further chemotherapy regimen, including EGFR-tyrosine kinase inhibitor, following this LDM regimen.

### Nail alterations

**Figure 1:**
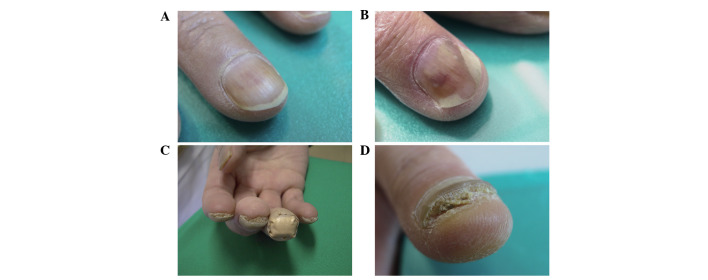
Representative images of nail alterations. (A) Dark pigmentation of fingernails (level 1); (B) onycholysis of fingernails (level 2); (C and D) subungual hyperkeratosis in fingernails (level 3).

No nail alterations were observed at the beginning of this LDM regimen. In the present study, various nail alterations were observed in 17 out of 49 patients (34.7%) during and after treatment. As previously described, docetaxel is known to induce various nail alterations. Examples include nail discoloration (including subungual hemorrhage), onycholysis, subungual hyperkeratosis, aseptic subungual abscess, loss of transverse line and Beau’s line (1). In this study, dark nail discoloration, onycholysis, subungual hyperkeratosis and aseptic subungual abscess were observed (Fig. 1), but Beau’s line and loss of transverse line were not. The metronomic schedule, as opposed to the conventional schedule of the maximum tolerated dose every three weeks, may be responsible for the observed spectrum of nail alterations. Nail alterations developed 8 weeks from the first administration of docetaxel. A spontaneous remission of nail alterations was observed after the withdrawal of LDM docetaxel treatment. Recovery of nail discoloration and reattachment of the nail to the nail bed was observed ∼8 weeks after the last docetaxel administration. These findings indicate that nail alterations due to the LDM docetaxel treatment are reversible.

**Figure 2:**
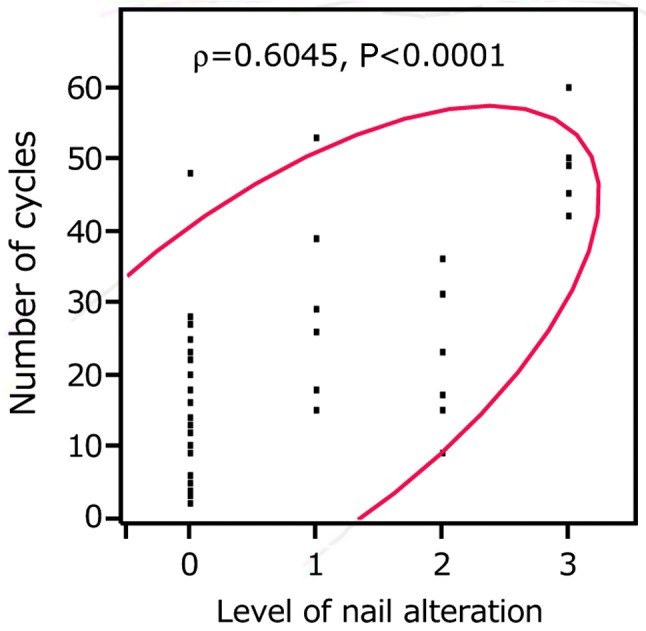
Correlation between the number of docetaxel administration cycles and the level of nail alterations. Scattergram with 95% probability ellipse showing significant correlation between the number of cycles and the level of nail alterations (R=0.6627, ϱ=0.6045, P<0.0001).

The nail alterations appeared to develop in a step-wise manner. Nail discoloration occurred first, followed by onycholysis. Finally, subungual hyperkeratosis and aseptic pus were observed. Since the most recent version of the common toxicity criteria (CTCAE v.4.03) for nail alterations is abstract and has only a few grades, a novel grading scale for nail alterations was developed in this study (16). Nail alterations were classified into 4 subsets according to severity (Table II): No evident nail alterations (level 0, 32 cases), nail discoloration only (level 1, 6 cases), nail discoloration with onycholysis (level 2, 6 cases) and nail discoloration and onycholysis with subungual hyperkeratosis (level 3, 5 cases). Subsequently, we attempted to determine whether the cycle number of docetaxel administration correlated with a particular level of nail alterations. Spearman’s correlation coefficients (2-tailed) were used to evaluate the correlation between cycle number and the level of nail alterations. Statistical analysis revealed that a larger number of docetaxel administrations correlated with a higher degree of nail alterations (Spearman’s ϱ=0.6409, P<0.0001; Fig. 2).

### Risk factors

The risk factors for the development of nail alterations were assessed. Univariate analysis showed that the number of treatment cycles (P<0.0001), female gender (P=0.0212), PS 0 (P=0.0351) and treatment-associated pleural effusion (P=0.0058) were statistically significant risk factors for the development of nail alterations (Table I). However, multiple logistic regression analysis indicated that the number of treatment cycles [P=0.0005; odds ratio (OR), 1.148/cycle], PS 0 (P=0.0195; OR, 8.907) and treatment-associated pleural effusion (P=0.0492, OR, 10.138) were independent risk factors for the development of nail alterations (Table III).

### Effect of nail alterations on survival

**Figure 3:**
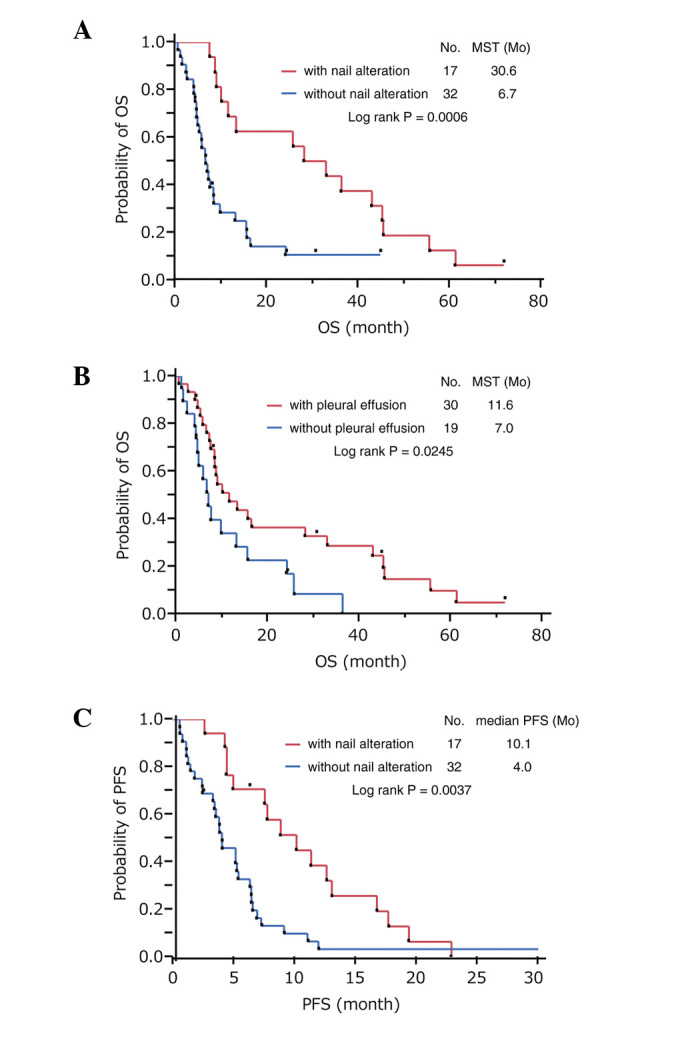
Kaplan-Meier survival curves for the patients who received low-dose metronomic docetaxel chemotherapy. Kaplan-Meier survival curves of overall survival (OS) for the patients classified according to (A) nail alterations and (B) pleural effusion. (C) Kaplan-Meier survival curves of progression-free survival (PFS) for the patients, classified according to nail alterations. MST, median survival time.

The effect of nail alterations in this LDM regimen (Table IV) on survival was evaluated. The MST for the patients with and without nail alterations was 30.6 and 6.7 months, respectively. Univariate analysis indicated that the patients with nail alterations showed significantly longer survival than those without nail alterations (P=0.0006; Fig. 3A). Additionally, female gender (P=0.0154), age <70 years (P=0.0108), the presence of adenocarcinoma (P=0.0049), prior chemotherapy (P=0.0099), PS 0 (P=0.0400) and treatment-associated pleural effusion (P= 0.0245) were significant prognostic factors for OS (Fig. 3B). By contrast, multivariate analysis revealed that the development of nail alterations [hazard ratio (HR), 0.2138; 95% CI, 0.0809–0.5515; P=0.0014) and prior systemic chemotherapy (HR, 0.3485; 95% CI, 0.1613–0.7459; P=0.0068) were independent favorable prognostic factors for OS. The patients with nail alterations also showed a significantly longer PFS than those without nail alterations (P=0.0037; Fig. 3C).

## Discussion

LDM chemotherapy targets the tumor vascular system (7–9). This suggests that the deterioration of constitutive remodeling of the microvascular network surrounding the tumor tissue may lead to a decrease in tumor burden. However, this type of chemotherapy may also damage non-tumor-associated microvascular networks in normal tissue, as tumor vessels are derived from the normal vascular system and composed of genetically normal endothelial cells. Microvessels in the nail bed or pleural architecture are also likely be affected. Consequently, nail alterations or capillary leakage resulting in pleural effusion may be induced. This may explain the close correlation observed in this study between nail alterations and pleural effusion. If a common pathogenic procedure is introduced by LDM chemotherapy, one might become a surrogate marker for the other. In this study, we showed that the development of nail alterations during LDM docetaxel chemotherapy was an independent favorable prognostic factor for OS. Therefore, nail alterations observed during treatment may be a potential surrogate marker for the efficacy of LDM docetaxel chemotherapy.

LDM chemotherapy has been shown to induce nail alterations more frequently than other treatments, particularly onycholysis, which is characterized by nail plate detachment from the nail bed and often results from subungual hemorrhage. In conventional chemotherapy with docetaxel (100 mg/m^2^ every 3 weeks), onycholysis was identified in 19% of patients (17). By contrast, onycholysis was observed in 12 out of 49 patients (24.5%) treated with a metronomic low dose of docetaxel (15 mg/m^2^ every week). Onycholysis and subungual hyperkeratosis have also been observed in patients with psoriasis, onychomycosis and certain autoimmune diseases, in addition to malignant tumor patients receiving chemotherapy (18–20). Although their pathological etiology remains to be elucidated, a number of studies support the possibility of a neurological involvement in the pathogenesis of these nail alterations. It has been reported that onycholysis or subungual hyperkeratosis does not extend to the nails of the paretic limb due to cerebrovascular events or metastatic brain tumors (21–24). These findings suggest that the neurological network is likely to play an essential transmissive role in the pathogenesis of nail alterations.

Another common pathogenic process for nail alterations may be capillary leakage, an alternative antitumor effect due to LDM docetaxel chemotherapy. A few studies have indicated that certain neuropeptides are able to induce an increase in vascular permeability and consequent plasma leakage (25,26). Moreover, docetaxel enhances microtubule assembly, leading to an interference of axonal flow and an impediment to the innervation of the microvascular network (27,28). According to these findings, treatment-associated adverse neurological effects may also be a favorable predictive factor for LDM docetaxel chemotherapy. However, no survival benefit was able to be associated with neurotoxicities in this study, as no neurological events occurred in our study population. Additionally, no significant results have been reported to support the effect of neurotoxicities associated with taxane-based chemotherapy on survival. A possible explanation for this is that neurosensory and neurovascular networks might be independently affected by taxanes. A recent study showed that there was no association between intraepidermal nerve fiber density and neurological symptoms during chemotherapy with neurotoxic agents (29).

Although we have clearly demonstrated the favorable impact of treatment-associated nail alterations on survival and shown for the first time that these nail alterations act as a surrogate marker for the efficacy of LDM docetaxel chemotherapy, this study had a number of limitations. The study population was small and heterogeneous, containing previously untreated and relapsed patients. In addition, the arbitrary nail alteration severity index defined in this study was qualitative, not quantitative.

The quality of life for patients undergoing antineoplastic treatment is becoming a more important issue. The quality and length of life for an individual should not be considered in mutually exclusive terms. Numerous physicians have pointed out that nail alterations are not a life-threatening adverse effect and are therefore not enough of a reason to abstain from chemotherapy (13). In the present study, we have demonstrated for the first time that nail alterations may act as a surrogate marker for the efficacy of LDM chemotherapy. Our preliminary findings provide a basis for further investigation into LDM chemotherapy.

## Figures and Tables

**Table I:** Patient and disease characteristics.

Characteristics	Total patients (n=49)	Patients with nail alterations (n=17)	Patients without nail alterations (n=32)	P-value
Gender				0.0212^a^
Male	39	10	29	
Female	10	7	3	
Age (years)				0.8457
Median	69	71	68.5	
Range	32–85	49–85	32–83	
ECOG PS				0.0351^a^
0	21	11	10	
1	28	6	22	
Histology (cytology)				0.1347
Ad	30	13	17	
Sq	19	4	15	
Disease stage				1.0000
III	22	8	14	
IV	27	9	18	
Prior systemic therapies				0.5480
0 regimen	22	8	19	
≥1 regimen	27	9	13	
Treatment cycles	1,003	557	446	<0.0001^a^
Median	16	31	12	
Range	2–60	9–60	2–48	
DTX dose (mg/body/week)				0.5073
Median	20	20	20.5	
Range	17–28	17–27	18–28	
Post-systemic therapies				0.1562
0 regimen	38	11	27	
≥1 regimen	11	6	5	
Pleural effusion				0.0058^a^
Observed	30	15	15	
Not observed	19	2	17	
ORR (%)	8.2	17.6	3.1	0.1139
DCR (%)	53.1	70.6	43.8	0.1317

a P<0.05. ECOG, Eastern Cooperative Oncology Group; PS, performance status; Ad, adenocarcinoma; Sq, squamous cell carcinoma; DTX, docetaxel; ORR, overall response rate; DCR, disease control rate.

**Table II:** Summary of nail alterations observed (n=49).

Level	Definition	Patients, n (%)
0	No evident nail alterations	32 (65.3)
1	Discoloration	6 (12.2)
2	Level 1 + onycholysis	6 (12.2)
3	Level 2 + subungual hyperkeratosis	5 (10.2)
1–3	Total nail alterations	17 (34.7)

**Table III:** Multiple logistic regression analysis of the clinical parameters associated with nail alteration.

Clinical parameters	OR (95% CI)	P-value
Number of cycles (/cycle)	1.148 (1.053–1.296)	0.0005^a^
Female vs. male	8.736 (0.986–128.278)	0.0515
PS 0 vs. PS 1	8.907 (1.393–97.050)	0.0195^a^
Sq vs. Ad	3.450 (0.381–49.322)	0.2803
Pleural effusion vs. absent	10.138 (1.007–253.901)	0.0492^a^

a P<0.05. OR, odds ratio; 95% CI, 95% confidence interval; Sq, squamous cell carcinoma; Ad, adenocarcinoma; PS, performance status. R^2^=0.5101, P<0.0001.

**Table IV:** Univariate and multivariate analysis for OS.

	Univariate analysis		Multivariate analysis	
Covariate	MST (Mo)	P-value	HR (95% CI)	P-value
Female vs. male	36.4 vs. 8.5	0.0154^a^	1.0949 (0.3495–2.9692)	0.8673
Age <70 vs. ≥70 years	16.4 vs. 7.5	0.0108^a^	0.6855 (0.3262–1.4259)	0.3107
Ad vs. Sq	16.4 vs. 7.2	0.0049^a^	0.6409 (0.3146–1.3271)	0.2266
Relapsed vs. naïve	16.4 vs. 7.6	0.0099^a^	0.3485 (0.1613–0.7459)	0.0068^a^
PS 0 vs. PS 1	25.7 vs. 7.6	0.0400^a^	0.9548 (0.4395–2.0791)	0.9065
Stage III vs. IV	9.0 vs. 9.8	0.3758	0.7655 (0.3792–1.5090)	0.4424
Nail alterations (observed vs. absent)	30.6 vs. 6.7	0.0006^a^	0.2138 (0.0809–0.5515)	0.0014^a^
Pleural effusion (observed vs. absent)	11.6 vs. 7.0	0.0245^a^	0.5613 (0.2908–1.0860)	0.0858

a P<0.05. OS, overall survival; MST, median survival time; Mo, months; HR, hazard ratio; 95% CI, 95% confidence interval; Ad, adenocarcinoma; Sq, squamous cell carcinoma; PS, performance status.
